# Autoimmune Cytopenias in Chronic Lymphocytic Leukemia: Focus on Molecular Aspects

**DOI:** 10.3389/fonc.2019.01435

**Published:** 2020-01-10

**Authors:** Bruno Fattizzo, Wilma Barcellini

**Affiliations:** ^1^Fondazione IRCCS Ca' Granda Ospedale Maggiore Policlinico, University of Milan, Milan, Italy; ^2^Fondazione IRCCS Ca' Granda Ospedale Maggiore Policlinico, Milan, Italy

**Keywords:** autoimmune hemolytic anemia, immune thrombocytopenia, chronic lymphocytic leukemia, Evans' syndrome, molecular

## Abstract

Autoimmune cytopenias, particularly autoimmune hemolytic anemia (AIHA) and immune thrombocytopenia (ITP), complicate up to 25% of chronic lymphocytic leukemia (CLL) cases. Their occurrence correlates with a more aggressive disease with unmutated VHIG status and unfavorable cytogenetics (17p and 11q deletions). CLL lymphocytes are thought to be responsible of a number of pathogenic mechanisms, including aberrant antigen presentation and cytokine production. Moreover, pathogenic B-cell lymphocytes may induce T-cell subsets imbalance that favors the emergence of autoreactive B-cells producing anti-red blood cells and anti-platelets autoantibodies. In the last 15 years, molecular insights into the pathogenesis of both primary and secondary AIHA/ITP has shown that autoreactive B-cells often display stereotyped B-cell receptor and that the autoantibodies themselves have restricted phenotypes. Moreover, a skewed T-cell repertoire and clonal T cells (mainly CD8+) may be present. In addition, an imbalance of T regulatory-/T helper 17-cells ratio has been involved in AIHA and ITP development, and correlates with various cytokine genes polymorphisms. Finally, altered miRNA and lnRNA profiles have been found in autoimmune cytopenias and seem to correlate with disease phase. Genomic studies are limited in these forms, except for recurrent mutations of KMT2D and CARD11 in cold agglutinin disease, which is considered a clonal B-cell lymphoproliferative disorder resulting in AIHA. In this manuscript, we review the most recent literature on AIHA and ITP secondary to CLL, focusing on available molecular evidences of pathogenic, clinical, and prognostic relevance.

## Introduction

The impact of autoimmune cytopenias (AIC) complicating chronic lymphocytic leukemia (CLL), particularly autoimmune hemolytic anemia (AIHA) and immune thrombocytopenia (ITP) is variable, ranging from mild asymptomatic cytopenias case without indication to CLL treatment, to severe transfusion dependent patients with abrupt onset and CLL progression. Each patient needs to be carefully evaluated, since the different pictures require a specific approach. Given this heterogeneity, the variability of response to immune-suppression, and the possible association/development of clonal diseases (lymphoproliferation or myelodysplasia), the genomic landscape of AIC is of particular interest.

In this manuscript, we will review the most recent literature on AIHA and ITP secondary to CLL with a brief summary of their clinical management. In particular we will focus on available molecular evidences of pathogenic, clinical, and prognostic relevance.

## Epidemiology and Pathogenesis

**Figure 1:**
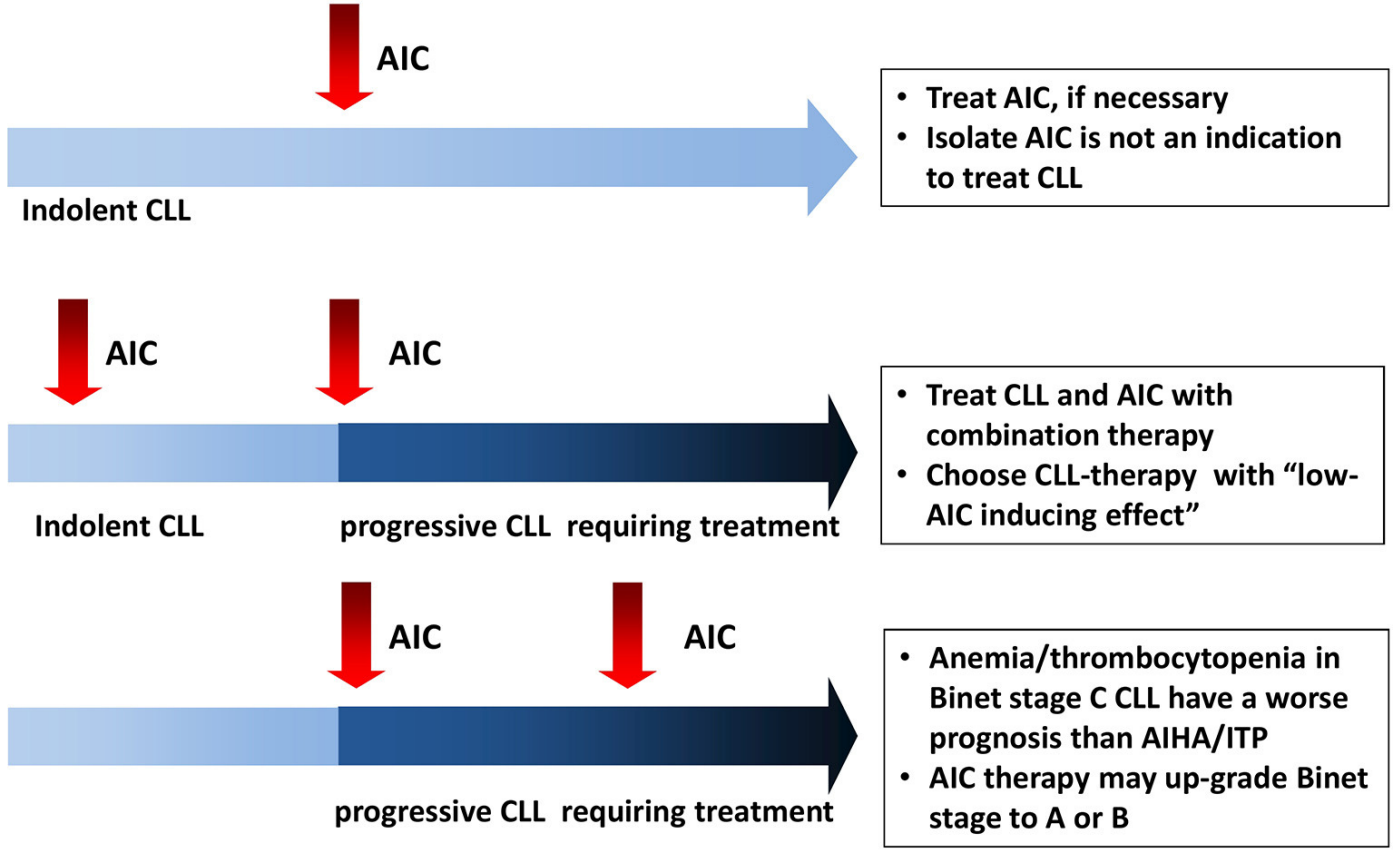
Autoimmune cytopenias (AIC) in chronic lymphocytic leukemia (CLL): the heterogeneity of onset imposes different management in each context.

AIC may complicate CLL course at any time, from diagnosis to disease progression (Figure 1) (1). AIHA are the most frequent form (7–10% of cases), followed by ITP (1–5%), and rarer entities such as pure red cell aplasia (PRCA, <1%) and autoimmune granulocytopenia (AIG 0.17%). From a pathogenic point of view, CLL associated AIC are mediated by a complex orchestration of humoral, cellular, and innate immunity: (1) IgG auto-antibodies coat erythrocytes, platelets, and neutrophils with consequent antibody-dependent cellular cytotoxicity and complement-mediated destruction in the reticuloendothelial system (spleen and liver) or in the blood stream. (2) Anti-erythroblast and megakaryocyte autoantibodies can impair bone marrow compensatory response. (3) Autoreactive T-cells produce inflammatory cytokines and further inhibit myelopoiesis. (4) Natural killer cells have been shown to destroy erythroblasts from CLL patients *in vitro*, confirming a role for innate immunity.

As regards autoantibodies, they are polyclonal high-affinity IgG produced by non-malignant self-reactive B-cells in 90% of cases. CLL cells may also produce autoantibodies (mainly IgM) in <10% of cases (2–5), and have been shown to secrete soluble factors inducing a dysregulation of bone marrow microenvironment (6, 7). Further pathogenic mechanisms, are the direct antigen presentation by CLL cells that may induce self-reactive T helper cells, and the production of non-functional T regulatory cells (T-regs) (8–10). The latter become unable to eliminate non-neoplastic autoreactive T- and B-cells leading to autoimmune phenomena (11–14). In addition, an increased incidence of autoimmune cytopenias in CLL is associated to an imbalance in the ratio between Th17 cells and T-regs (15). Finally, CLL patients developing autoimmune phenomena displayed a reduction of Toll-like receptors (TLR)-4, an important player of the innate immunity, together with a lower expression of TLR2, and an increase of TLR7, TLR9, and TLR10 (16–18).

### Influence of CLL Therapy on the Development of AIC

The influence of CLL therapy on the development of AIC deserves special consideration: single-agent purine analogs (i.e., fludarabine) may induce CLL-AIHA (19, 20) possibly worsening the imbalance between Th17 and T-regs (21). FC and FCR combination schemes (fludarabine, cyclophosphamide, and rituximab) in the CLL8 trial (22) showed very low incidence (<1%) of hemolytic anemia, as did bendamustine rituximab (BR) association (even if anecdotic PRCA cases have been described) (23). Alemtuzumab led to treatment-emergent ITP in 9% of CLL cases (24), again possibly due to T-cell dysregulation. Concerning small molecules, the most interesting data are available for Bruton's tyrosine kinase inhibitor ibrutinib: new-onset AIC was rarely reported in the largest studies performed so far (25–27). Moreover, AIC resolution occurred in about a half of CLL-AIC patients (*N* = 13) (26) and most CLL-AIC cases were able to discontinue AIC-therapy after a median of 4.7 months (*N* = 301 of whom 7% with ongoing AIC therapy) (27). Similar data were reported in a more recent study of 193 patients: 67% of 29 cases with AIC pre-ibrutinib could discontinue/taper AIC treatment and new-onset AIC occurred in 6% (all with unmutated IGHV) (28). Recent evidences suggest an inhibitory role of ibrutinib on autoreactive T cells, through interleukin-2-inducible kinase (ITK)suppression, leading the way for its use in T-cell mediated autoimmune conditions (i.e., graft vs. host disease) (29). Regarding other small molecules, limited data are available for idelalisib (that targets phosphoinositide 3-kinase), and venetoclax (a BCL-2 antagonist), although the presence of autoimmune phenomena was an exclusion criteria in various trials. Concerning venetoclax, it has been reported to be associated to the occurrence, although rarely, of AIHA in large CLL registrative trials (30). Interestingly, increased incidence of autoimmune complications (hepatitis, colitis, and pneumonitis) has been reported for idelalisib (31, 32).

## Management of Autoimmune Hemolytic Anemia Secondary to CLL

### Diagnosis

Management of AIHA in CLL requires the evaluation and exclusion of the other possible causes of anemia, including bone marrow infiltration/failure, bleeding, vitamin or iron deficiencies, and renal disease. As previously suggested, a diagnosis of AIHA can be established in the presence of Hb <11 g/dL, no chemotherapy in the previous month, variable alteration of hemolytic markers (increased unconjugated bilirubin, elevated lactate dehydrogenase, consumption of haptoglobin, increased absolute reticulocyte counts), and the positivity of the direct antiglobulin test (DAT) (1, 33). The latter allow to distinguish warm (wAIHA: DAT positive for IgG or IgG+C3d at low titer and negative autoagglutination at 20°C) from cold (cAIHA) cases (DAT positive for C3d and positive autoagglutination at 20°C). Of note, CLL itself may be a confounder in the differential diagnosis, since LDH may be elevated during disease progression, haptoglobin increased due to chronic/acute inflammation, and reticulocytosis may be absent or inadequate due to bone marrow infiltration or suppression by cytokine storm and/or anti-erythroblasts antibodies (1). The latter, demonstrated in a proportion of CLL cases through the mitogen-stimulated DAT, were associated to increased IL-4 and IFN-γ production, and may contribute to ineffective erythropoiesis (34). Furthermore, DAT positivity does not necessarily mean AIHA and in a longitudinal study of DAT+CLL cases only one third developed clinically overt hemolysis (35). Conversely, DAT negative AIHA cases may also be present (36), possibly due to the low-affinity or to the very small number of autoantibodies. In this context, the use of more sensitive techniques (microcolumn and solid-phase tests, or mitogen-stimulated DAT) may be useful (34). Finally, Bone marrow biopsy is usually necessary to document CLL infiltration and to rule out other causes (including bone marrow failure).

### Treatment

As regards therapy (Table 1), the acuteness of onset, the severity of the anemia and the degree of hemolysis should be considered, together with patient' symptoms, age and comorbidities. Blood transfusions are usually indicated if Hb < 6 g/dL or higher in elderly comorbid patients. Over-transfusion should be avoided since it carries high risk of allo-immunization. In CLL-cases, given underlying bone marrow impairment and inadequate reticulocytosis, transfusion requirement may be higher than in primary cases. Moreover, the evaluation of endogenous erythropoietin (to be performed before repeated transfusions that may confound the picture) could suggest the use of recombinant erythropoietin. For warm AIHA, steroid therapy is considered the first line (usually prednisone at 1 mg/kg day for 3–4 weeks, followed by a slow tapering in a total of 6 months). Methylprednisolone boli (2–10 mg/Kg day for 3 days) may be considered, with or without intravenous immunoglobulins (0.4 g/kg for 5 days or 1 g/kg for 2 days), in patients with acute hemolysis and slow response to steroid therapy (1, 37). The fewer patients with cAIHA may have a milder clinical presentation with Hb levels >9 g/dL and cold agglutinin associated symptoms (acrocyanosis, itch, urticarial, etc.) and may require a watchful waiting approach. Treatment should be reserved for transfusion-dependent cases, active hemolysis (even if increase of LDH is difficult to judge in CLL), and invalidating cAIHA symptoms. Corticosteroids are usually effective only at high doses, and are a useful tool only in the acute setting. Prompt rituximab treatment should be considered, together with a quick steroid tapering after Hb stabilization. Rituximab is currently considered the first therapy line in cAIHA at standard dose of 375 mg/sm weekly for 4 weeks, with an overall response in up to 70–100% of patients (1, 39, 44). Considering patients refractory to first-line treatment (both wAIHA and cAIHA), current guidelines advice the introduction of a CLL directed therapy. The choice between chemoimmunotherapy and small molecules should be made according to current guidelines (patient age/comorbidities and CLL molecular characteristics) and considering potentially hemolytic side effects (avoid fludarabine single agent). As regards published studies specifically addressing refractory CLL-AIHA, rituximab in various combinations was able to induce high (>80%) and durable response rates: 89% (*N* = 8) with cyclophosphamide and dexamethasone (RCD) (54, 55), 95% (*N* = 20) with cyclophosphamide, vincristine, and prednisone (R-CVP) (56), and 80% with bendamustine (*N* = 26), with a median relapse free survival of 28 months (45, 46). Good results have also been reported in association with oral fludarabine, even if mainly in primary cAIHA cases (47). The only exception to this aggressive approach regards steroid-refractory wAIHA with no signs of CLL progression. In this setting, a possible strategy is to administer rituximab single agent with a reported efficacy in 72% of cases, of whom 40% sustained responses at 17 months (38, 39). Alemtuzumab has been abandoned because of serious infectious and autoimmune complications, as also happened for splenectomy (41–43). Cytotoxic immunesuppressors showed heterogeneous and weak efficacy in primary AIHA and are usually not administered in CLL secondary cases (40, 58). New generation monoclonal antibodies, such as of atumumab and obinutuzumab, may also be useful in secondary AIHA (59). As cited above, ibrutinib seems to be safe in patients with CLL-AIHA and progressive disease, and a phase II trial of ibrutinib combined to rituximab is ongoing in CLL-wAIHA [NCT03827603]. Regarding venetoclax, case reports of successful treatment have been published (60, 61).

**Table 1:** Specific therapies and relative outcomes for warm and cold autoimmune hemolytic anemia and immune thrombocytopenia secondary to chronic lymphocytic leukemia (CLL).

**Treatment**	**Line**	**Overall response rate %**	**References**
**WARM AUTOIMMUNE HEMOLYTIC ANEMIA wAIHA**			
**Prednisone**1 mg/kg/day for 3–4 weeks	1st	84–90	(1, 37)
**Dexamethasone**40 mg/day for 4 days, 2–6 cycles every 2–4 weeks	1st	100	
**Rituximab**375 mg sqm weekly × 4	2nd or >	72–80	(38, 39)
**Cyclosporine**3–5 mg/Kg day	3nd or >	56	(40)
**Alemtuzumab**30 mg × 3/week × 4–12 weeks	3nd or >	100	(41, 42)
**Splenectomy**	3nd or >	69–78	(43)
**COLD AUTOIMMUNE HEMOLYTIC ANEMIA cAIHA**			
**Rituximab**375 mg/sqm weekly × 4	1st	50–70	(1, 39, 44)
**Rituximab+Bendamustine**90 mg/sqm	2nd or >	71–80	(45, 46)
**Rituximab+Fludarabine**40 mg/sqm	2nd or >	76	(47)
**IMMUNE THROMBOCYTOPENIA ITP**			
**Prednisone**1 mg/kg/day for 3–4 weeks	1st	90	(37)
**Dexamethasone**40 mg/day for 4 days, 2–6 cycles every 2–4 weeks	1st	90	
**Rituximab**375 mg sqm weekly × 4	2nd or >	78	(48–50)
**TPO analog**Romiplostim 1–10 mcg/Kg week Eltrombopag 50–150 mg day	3rd or >	80	(51–53)
**Alemtuzumab**30 mg × 3 week × 4–12 weeks	3rd or >	100	(42)
**Cyclosporine**3–5 mg/Kg day	3rd or >	62	(40)
**Splenectomy**	3rd or >	61	(43)
**Other rituximab associations reported for warm and cold AIHA, and ITP**			
**Rituximab+cyclophosphamide and dexamethasone (RCD)**	2nd or >	89	(54, 55)
**Rituximab+cyclophosphamide, vincristine, and prednisone (R-CVP)**	2nd or >	95	(56, 57)

*Current guidelines suggest CLL-directed therapy in relapsed/refractory cases*.

## Management of Immune Thrombocytopenia Secondary to CLL

### Diagnosis

The same diagnostic caveats mentioned for CLL-AIHA have to be considered in the thrombocytopenic patient. ITP should be suspected in a CLL patient with <100 × 10^9^/L platelets, with no chemotherapy in the previous month; moreover signs of CLL progression should be excluded (progressive splenomegaly, concomitant anemia, significant bone marrow CLL infiltrate, evidence of bone marrow failure/dysplasia). Other secondary causes (infections, drug-induced thrombocytopenia, thrombotic microangiopathies, and heparin-induced thrombocytopenia) should also be ruled out. Antiplatelet antibodies are of little aid due to the low sensitivity and specificity of the test, and usually not performed (1).

### Treatment

ITP should be treated only in case of severe thrombocytopenia (Plt < 30 × 10^9^/L) or bleeding. First-line therapy with steroids (prednisone at 1 mg/kg day for 1 month, followed by a slow tapering, or dexamethasone 40 mg/day × 4 days 1–3 cycles) is the standard approach, with about 50% responders. Intravenous immunoglobulin can be added in case of bleeding or slow response to steroids, again with 50% response rate [(27)]. Platelet transfusion may be required in case of life-threatening hemorrhage. Similarly to CLL-AIHA, steroid refractory cases would deserve CLL-directed therapy evaluation. Rituximab monotherapy was shown effective in 86% of CLL-ITP cases (57% complete response) (48), with 21 months response duration (49, 50). Rituximab combined to cyclophosphamide and dexamethasone or to cyclophosphamide, vincristine and prednisone had a high rate of durable responses in published experiences (55, 57). Splenectomy is usually discouraged given the increased infectious risk, older age and comorbidities of CLL patients. Finally, thrombopoietin mimetics (romiplostin and eltrombopag), indicated in refractory primary ITP, have shown high (up to 80%) and durable responses in patients with CLL-ITP (51–53, 62).

## Molecular Aspects in Primary and Secondary AIHA

Table 2 shows available studies addressing molecular aspects of warm and cold AIHA, both primary and secondary to lymphoproliferative disorders.

**Table 2:** Molecular findings in primary and secondary autoimmune hemolytic anemia (AIHA) and Evans' syndrome.

**Disease**	**Gene/Pathway**	**No. of patients**	**Technique**	**Impact and significance**		**References**
**PRIMARY AIHA**						
Cold AIHA	IGHV4-21	2	Nucleotide sequence analysis	Pathogenic	VH4-21 gene segment is responsible for the major cross-reactive idiotype	(63)
Cold AIHA	IGHV region	–	Nucleotide sequence analysis	Pathogenic	Specific IGVH regions are related to anti- i and I red blood cell antigens autoantibodies	(64)
Cold AIHA	IGHV4-34	–	PCR	Pathogenic	Anti-RBC antibodies are clonally restricted	(65)
Cold AIHA	IGHV3-23	–	Selection of phage-antibody library on human red cells	Pathogenic	//	(66)
Cold AIHA	+3 and +12	–	Chromosome analysis	Pathogenic	Autoreactive B-cells are clonal	(67, 68)
AIHA	TNF-α, LT-α, IL-10, IL-12, CTLA-4	17	PCR and specific restriction enzyme digestion	Pathogenic/therapeutic	AIHA show higher frequency of LT-α (+252) AG phenotype	(69)
Cold AIHA	IGKV3-20 and IGKV3-15	27	IGH and IG light chain gene sequencing	Pathogenic/therapeutic	IGHV and IGKV correlate with cold agglutinin disease onset and activity	(70)
AIHA	TCRG and TCRB	33	DNA sequencing	Pathogenic/therapeutic	Pathogenic T-cells are clonally restricted in AIHA	(71)
Cold AIHA	KMT2D and CARD11	16	Exome sequencing, targeted sequencing, Sanger sequencing	Pathogenic/therapeutic	Autoreactive B-cells display somatic mutations favoring proliferation	(72)
**SECONDARY AIHA**						
AIHA in CLL	IGVH51p1	12	PCR	Pathogenic	CLL patients expressing IGVH51p1 are more prone to AIHA	(73, 74)
AIHA in CLL	IGHV1-69, IGHV3-11, IGHV4-59, HCDR3	319	RT-PCR	Pathogenic/prognostic	Sterotyped heavy chains mutational status in CLL developing AIHA	(75)
AIHA primary/CLL and ITP	CTLA-4 exon 1	110	PCR	Pathogenic/prognostic/ therapeutic	CTLA-4 signaling is defective in AIHA, particularly in CLL cases	(76)
AIHA in CLL	miRNA−19a,20a,29c,146b-5p,186,223,324-3p,484,660	n.a.	RT-PCR	Pathogenic	Nine miRNA are preferentially expressed in CLL developing AIHA	(77)
AIHA in CLL	HCDR3 subset #3	585	PCR	Pathogenic/prognostic/ therapeutic	Sterotyped B-cell receptor subsets correlate with AIHA development	(78)
**PRIMARY AND SECONDARY EVANS' SYNDROME**						
Evans in CLL	IGHV	25	PCR	Pathogenic/prognostic	Majority of ES-CLL cases display stereotyped B cell receptor	(79)
AIHA and ITP	Fc-γ-R IIa and IIIa on red pulp macrophages	82	CFM and mRNA transcript analysis	Pathogenic/therapeutic	Spleen red pulp macrophages display distinct FC-γ-R expressions	(80)
AIHA and Evans in CLL	miR-150 and c-Myb	35	RT-PCR	Pathogenic	c-Myb expression is high and miR-150 is low in active hemolysis and correlate with Hb, bilirubin, and C3 levels	(81)
Pediatric Evans Syndrome	TNFRSF6, CTLA4, STAT3, PIK3CD, CBL, ADAR1, LRBA, RAG1, and KRAS	203	Sanger sequencing in 203; targeted NGS (tNGS) of 203 genes in 69 negative at Sanger (*n* = 69); whole-exome sequencing in selected cases	Pathogenic/prognostic/ therapeutic	Majority of pediatric ES display somatic mutations found in immune-deficiencies	(82)

*IGHV, immunoglobulin heavy chain variable region; +3 and +12, trisomy of chromosome 3 and 12; TNF-α, tumor necrosis factor alpha; LT-α, lymphotoxin alpha; IL-10 and -12, interleukin-10 and -12; CTLA-4, cytotoxic T-lymphocyte antigen-4; IGKV, immunoglobulin K light chain variable region; TCRG, T-cell receptor gamma; TCRB, T-cell receptor beta; miRNA, microRNA; Fc-γ-R, Fc-gamma-receptor; CFM, cytofluorimetry; PCR, polymerase chain reaction; RT-PCR, real time PCR; HCDR3, heavy chain domain region 3; ES, Evans syndrome; wAIHA and cAIHA, warm and cold autoimmune hemolytic anemia; ITP, immune thrombocytopenia; CLL, chronic lymphocytic leukemia; NGS, next generation sequencing*.

### Studies on Immunoglobulin Genes

Since the autoantibody is the major pathogenic player, the larger and older experiments focused on the configuration of the genes of the variable region of the immunoglobulin heavy chains (IGHV) encoding AIHA autoantibodies and demonstrated that some rearrangements are preferentially involved. Almost all patients with cAIHA displayed monoclonal antibodies encoded by the *IGHV4-34* gene, responsible for I antigen binding (63–65). Rarely, *IGHV3* family genes may also encode anti-I cold agglutinins, in particular *IGHV3-23* and *IGKV3-20* (66, 70, 83). Concerning Ig light chain genes, the *IGKV3-20* gene and the *IGHV3-15* gene are used in most cAIHA patients and contribute to I antigen binding. From a clinical perspective, mutations in the complementarity determining region (CDR)2 and in the framework region 3 (FR3) of *IGHV4-34* correlated with lower hemoglobin levels (70), whilst those in the *IGKV3-20* CDR3 correlated with younger age at diagnosis. These findings are in line with the clonal nature of cAIHA that is currently considered a distinct lymphoproliferative disorder, with some level of bone marrow infiltration morphologically different from other non-Hodgkin lymphomas. The presence of stereotyped light chains of cAIHA may be of therapeutic interest, since anti-light chain vaccinations with *IGKV3-20* are under investigation for lymphoproliferative diseases (84).

Other studies focused on B-cell receptor configuration and its contribution to AIC development. It is known that unmutated IGHV carries a strong prognostic impact on CLL course and correlates with a higher incidence of AIC (78, 85–89). The binding of auto-antigens to unmutated CLL cells activates a signal transduction (i.e., phosphorylation of SYK and ZAP-70) promoting survival and proliferation (90). More recently, a high recurrence of stereotyped IGHV aminoacid sequences has been observed in CLL patients developing AIC (91–95). Efremov et al. (73) reported an over-representation of the *51p1 VH* gene; in other two large studies (*N* = 319 and *N* = 585), patients developing AIHA showed a more frequent expression of unmutated *IGHV1-69, IGHV3-11, IGHV4-59, IGHV4–30, IGHD2-2, and IGHJ6* genes, unfavorable [del(17)(p13) and del(11)(q23)] cytogenetics, and stereotyped HCDR3 sequences (75, 78). Finally, stereotyped B cell receptor configuration was found in 66% of CLL secondary Evans syndrome, a known severe complication defined by the association of AIHA and ITP (79).

### Studies on Cell-Mediated Immunity

Since a T-cell imbalance is known to play a part in AIC development (higher Th17/T regulatory ratio, Th1 to Th2 cytokine shift, increased APC activity), other studies focused on T-cell compartment. They showed the presence of clonal T-cell populations, mainly CD8+, in about 50% of AIHA patients (*N* = 33), higher than in controls (71). Another study (76) evaluated cytotoxic T-lymphocyte antigen-4 (*CTLA-4*) gene status in patients with primary or secondary AIC (20 primary AIHA, 30 CLL-AIHA, and 60 ITP). *CTLA-4* is a negative regulator of T-cell responses and has been implicated in various autoimmune diseases (96, 97). A high prevalence of an A to G polymorphism at position 49 was found among AIHA cases, particularly in the CLL-AIHA group (73% vs. 47% in the control group), suggesting *CTLA-4* mediated T-cell imbalance in these cases. A more recent study found a significant higher frequency of lymphotoxin-α (LT-α) (+252) AG phenotype in 17 AIHA cases compared to controls (41% vs. 13%) (69). LT-α (also known as TNF-β), is involved in the regulation of cell survival, proliferation, differentiation, and apoptosis, and plays an important role in innate immune regulation and immune-surveillance (98).

Finally, it has been reckoned that AIHA clinical picture also depends on the level of the monocyte-macrophage system activation and some Authors studied FcγR subtypes expressions in various tissues in 82 AIHA cases. They found that red pulp macrophages predominantly expressed the low-affinity receptors FcγRIIa and FcγRIIIa, did not express the inhibitory FcγRIIb, and expressed very low levels of the high-affinity receptor FcγRI, compared to blood monocytes (80). This may be of therapeutic interest, given that FcγR and its signaling have recently become a target in autoimmune diseases.

### Genomic Studies

The use of advanced target and non-target sequencing assays offered further insights in AIHA pathogenesis. In particular, in a study of 16 primary cAIHA, next generation sequencing of bone marrow B-cells allowed the identification of recurrent mutations of *KMT2D* and *CARD11* in 69% and 31% of cases, respectively (72). Similar mutations have also been reported in lymphomas as well as in Kabuki syndrome, a congenital disorder characterized by malformations, immune-deficiency, and development of autoimmune diseases. Loss of *KMT2D* function increases B cell proliferation, impedes class switch recombination (99), and may concur to survival of autoreactive B cells synergizing with *IGHV4-34*-encoded immunoglobulin receptor stimulation (72). *CARD11* mutations were shown to induce constitutive activation of the NF-kB pathway, similarly to what observed in diffuse large B-cell lymphoma. Evaluation of *KMT2D* and *CARD11* might be of diagnostic utility in cAIHA, and would help to distinguish it from *MYD88* mutated lymphoplasmacytic lymphoma. Genomic studies may give hints for novel therapeutic approach. In fact, histone deacetylase inhibitors, that have been used in lymphoma, myeloma and Kabuki syndrome, might have a therapeutic potential in cAIHA with *KMT2D* mutations (72, 100). Similarly, therapies targeting *CARD11* gain-of-function mutations are under investigation for B cell lymphomas and may be studied also in cAIHA (101).

Another very recent study evaluated a large series of pediatric patients with Evans syndrome by Sanger sequencing, targeted NGS, and whole exome sequencing (*N* = 80): 65% received a genetic diagnosis, 49 had a germline mutation, and 3 somatic variants. Pathogenic mutations in genes involved in primary immunodeficiencies (*TNFRSF6, CTLA4, STAT3, PIK3CD, CBL, ADAR1, LRBA, RAG1*, and *KRAS*) were found in 40% of cases, and probable pathogenic variants in 16 genes not previously reported in autoimmune disease were detected in 25%. It was already known that children with primary immunodeficiency are more prone to develop immune cytopenia, whilst in adult Evans' syndrome a primary immunodeficiency was identified in 9% of cases only (102). In the pediatric study, mutated patients showed more severe disease with higher treatment requirement (>number of therapy lines) and mortality. These data confirm that a higher genomic burden is probably involved in pediatric cases, and that it seems to have prognostic and therapeutic significance (82). For instance, patients with autoimmune lymphoproliferative syndrome (ALPS), caused by germline and somatic *TNFRSF6* mutations, are more prone to develop severe persistent hypogammaglobulinemia after rituximab treatment, and splenectomy is contraindicated. Since rituximab is highly effective and broadly used in Evans syndrome, a prompt diagnosis of such cases is of great importance. Moreover, 36% of cases had potentially targetable mutations that will be suitable for new therapeutic approaches including rapamycin inhibitors (in ALPS or a *PIK3d* activation syndrome) (103, 104), CTLA-4 fusion protein (in *CTLA-4* and *LRBA* deficiency) (105, 106), JAK inhibitors (in patients with *JAK1* or *JAK2* mutations) (107), and calcineurin inhibitors (in patients with *NFATC1* variants) (108).

### Studies on MicroRNAs

MicroRNAs (miRNAs) are small single strain RNAs mainly implied in gene expression regulation at transcriptional and post-transcriptional level. They have been associated with different clinical-biological forms of CLL and are also known to play a substantial role in autoimmunity (77). In a recent study evaluating malignant B-cells from CLL-AIHA patients, nine down-regulated miRNAs were identified (i.e., miR-19a, miR-20a, miR-29c, miR-146b-5p, miR-186, miR-223, miR-324-3p, miR-484, and miR-660), of whom two (i.e., miR-20a and miR-146b-5p) known to be involved in autoimmune phenomena. Interestingly, miR-146b-5p was shown to modulate the expression of CD80, a molecule involved in the B-T cell synapse formation and in restoring the APC capacity of CLL cells. Another miRNA, miR-150, was recently studied in 35 patients with AIHA/Evans syndrome and was found low in patients with active hemolysis compared to those in remission or with CLL-AIHA. MiR-150 negatively correlated with bilirubin values and positively with Hb and complement levels, suggesting the role of miRNAs in predicting CLL evolution and treatment response (81).

## Molecular Aspects in Primary and Secondary ITP

### Studies on Immunoglobulin Genes

Similarly to AIHA, first molecular studies on primary ITP showed the presence of recurrent *IGHV* gene rearrangements in autoreactive B cells (Table 3) (109). Roark and Colleagues, found an association with rearrangements of *IGHV3-30*, and further reports showed that IGHV30 encoded IgM and IgG anti-GPIIb autoantibodies (122–125). Interestingly, *IGHV3-30* is highly employed also in AIHA, CLL, and immunodeficiencies and this may explain the association with ITP (74, 126). In CLL patients, it has been shown that the risk of developing ITP was higher among patients with stereotyped subset #1 (*IGHV1–5-7/IGHD6–19/IGHJ4*) and #7 (*IGHV1–69 or IGHV3–30/IGHD3-3/IGHJ6*) in HCDR3 region (78). Other IGHV involved in anti-platelets autoantibodies are *VH1-02, VH1-46, VH3-21*, and *VH4-59*. Interestingly, a specific heavy- and light-chain pairing seems to be necessary to enable antibody pathogenicity (127–131). Anti-platelets autoantibodies appear to share single heavy-chain VHDJH and have undergone isotype switching (hallmark of a T-cell-dependent, antigen-driven response). These aspects are not observed in naturally occurring anti-platelet antibodies that are polyreactive IgM with little or no somatic mutation of their variable regions, and are responsible for platelets turnover. The presence of stereotyped *IGHV* asset could be of therapeutic interest in ITP, since *IGHV3-30*-targeted reagents, such as anti-idiotypic antibodies derived from mice (132, 133) or humans (125) are under evaluation (134–137).

**Table 3:** Molecular findings in primary and secondary immune thrombocytopenia (ITP).

**Disease**	**Gene/Pathway**	**No. of patients**	**Technique**	**Impact and significance**		**References**
**PRIMARY AND SECONDARY ITP**						
ITP	IGVH3-30	2	PCR	Pathogenic/therapeutic	Anti-PLT antibodies are clonally restricted	(109)
ITP	CD41, c-Myb, c-MPL, caspase-2, caspase-9, GATA-1, Bcl-xl	Murine models	RT-PCR	Pathogenic	Hyperexpression of those genes in the spleen of ITP mice	
ITP	Haptoglobin	58	Matrix assested laser desorption/ionization time-of-flight mass spectrometry	Prognostic/predictive	High haptoglobin levels predict long-term response to splencetomy	(110)
ITP	Th17 associated signaling factors	–	–	Pathogenic	Neutralization of IL-17A and IL-21 regulates Treg/Th17 imbalance	(111)
ITP	STAT1	328	Sequenom Mass Array	Pathogenic	STAT1 rs1467199 SNP plays a role in IFN-γ dependent development of ITP	(112)
ITP	miRNA	32	RT-PCR	Pathogenic/therapeutic	44 miRNAs are differentially expressed in ITP pre- and post-QSBLE therapy	(113)
ITP	miRNA-125a-5p	30	RT-PCR	Pathogenic	lncRNA MEG3 inhibits miRNA-125a-5p favoring Treg/Th17 imbalance	(114)
Primary and secondary ITP	Proteomics	134	Surface-enhanced laser desorption/ionization time-of-flight mass spectrometry	Diagnostic	6 marker proteins distinguishing primary from secondary ITP	(115)
ITP	Bcl-6, c-Maf, Blimp-1, ICOSL, TACI, BAFFR	85	RT-PCR	Pathogenic	T follicular helper cells display different frequency and regulation between newly diagnosed and chronic pediatric ITP	(116)
ITP	TNFRSF13B	2	GEP and WES	Pathogenic	G76S mutation is a gain-of-function mutation and predispose to familial and sporadic ITP	(117)
ITP	IL-17F rs763780	165	RT-PCR	Pathogenic	IL-17F rs763780 G allele frequency is significantly lower in ITP vs. controls	(118)
ITP	NLRP3 inflammosome	403	RT-PCR	Pathogenic/therapeutic	NF-Kb-94ins/del ATTG genotype correlates with Th17 imbalance	(119)
ITP	Long non-coding RNAs	64	Microarray studies and RT-PCR	Pathogenic	lncRNAs are differentially upregulated/downregulated in newly-diagnosed and chronic ITP vs. healthy controls	(120)
ITP	Integrated mRNA and miRNA	4	Microarray technique and RT-PCR	Pathogenic	Cellular stress response is deregulated in mesenchymal stem cells from ITP cases	(121)

*ITP, immune thrombocytopenia; CLL, chronic lymphocytic leukemia; IGHV, immunoglobulin heavy chain variable region; PLT, platelets; Th17, T- helper 17 cells; Th1, T helper 1; IL-17 and -21, interleukin-17 and -21; lncRNA, long non-coding RNA; Treg, T regulatory cells; miRNA, microRNA; GEP, gene expression profiling; WES, whole exome sequencing; PCR, polymerase chain reaction; RT-PCR, real time PCR; NGS, next generation sequencing*.

### Studies on Cell-Mediated Immunity

Th17 are known to mediate autoimmunity through the release of pro-inflammatory cytokines (IL-2/IL-17). Th17 cells response, together with Th2 (anti-inflammatory), regulatory B (Breg), and Treg cells inhibition (with decrease in IL-10/TGF-β), favor ITP persistent/chronic phase. As a matter of fact, therapy with corticosteroids, rituximab, and thrombopoietin receptor agonists have all be shown to increase Tregs and TGF-β levels (TPO agonists also increase Breg). Given the importance of these cytokine dysregulation, some Authors focused on Treg/Th17 imbalance and on cytokine genes polymorphisms. In a recent study, it has been shown that *NF-*κ*B*-94ins/del ATTG genotype (involved in the *NLRP3* inflammasome) contributes to ITP development and to imbalanced Th17 cell response (119). Another study on *IL-17F* rs763780 polymorphism, that has been associated with IL-17 expression and activity, showed a lower prevalence in ITP cases (*N* = 165) compared to healthy controls (118). Finally, Hu et al. demonstrated that IL-17A and IL-21 are able to upregulate *STAT-1, STAT-3, STAT-5* or RAR-related orphan receptor C (*RORC*), resulting in decreased Treg/Th17 balance in newly diagnosed ITP cases. This imbalance recovered after ITP remission and was reversed by the neutralization of IL-17A or IL-21 through targeting antibodies (111). IL-21 levels, together with IL-4, were also found to be abnormal in pediatric ITP (*N* = 85), and to affect T follicular helper cells levels and regulation (116). IL-17A or IL-21 blockade could be a novel target for ITP.

### Studies on Inflammatory Cytokines

Interferon (IFN)-γ signaling and tumor necrosis factor (TNF) are highly implicated in ITP pathogenesis and provides a link between autoimmunity, inflammation, and bone marrow failure. A polymorphism in the signal transducer and activator of transcription 1 protein (STAT1) rs1467199 SNP, the main target of IFN-γ down-stream emerged in a study of 328 ITP children, and was differentially found between newly diagnosed and chronic patients (112). More recently, microarray studies showed that a huge number of long non-coding RNAs (lncRNAs) were significantly up-regulated or down-regulated in newly diagnosed and chronic ITP patients vs. healthy individuals. TNF and granulocyte macrophage colony-stimulating factor signaling were the most interested pathways. Interestingly, lncRNAs ENST00000440492, ENST00000528366, NR_038920, and ENST00000552576 were able to distinguish newly diagnosed from chronic ITP (120). Finally, Peng et al. used gene expression profiling analysis and whole-exome sequencing on samples from family members with ITP, sporadic ITP cases and healthy individuals and identified a potential pathologic p.G76S heterozygous mutation on the *TNFRSF13B* gene. Mutated cases had upregulated cytokine-cytokine receptor interaction, increased serum TNFα, IL-17α, IFNγ, and BAFF levels, and enhanced binding capacity of APRIL ligand to B cells. Moreover, B cells transfected with the G76S mutation could induce human megakaryocyte apoptosis *in vitro* (117).

### Studies on MicroRNAs

MiRNAs expression was also evaluated in ITP in various reports: molecular studies of bone marrow mesenchymal stem cells from ITP patients showed that 740 genes and 32 miRNAs were differentially expressed compared to controls and correlated with the presence of cellular growth defects and functional abnormalities. The latter seem to be due to impaired cellular stress response, unfolded protein response, and reduced DNA transcription (121). Burenbatu and Colleagues, identified 44 miRNAs that are differentially expressed in ITP patients before and after treatment with the Mongolian medicine Qishunbaolier (QSBLE). Interestingly, 25 from these 44 miRNAs are downregulated in ITP as compared to controls, and are restored after QSBLE exposure (113). Finally, reduced miR-125a-5p expression has been linked Treg/Th17 imbalance. Li et al. demonstrated that miR-125a-5p expression is inhibited by *MEG3* overexpression in ITP patients (*N* = 30). Interestingly, dexamethasone was able to reduce *MEG3* expression *in vitro*, thus restoring Treg/Th17 ratio (114).

### Proteomics

Proteomic studies found some clinical implications: screen of 64 primary and 70 secondary ITP cases using surface-enhanced laser desorption/ionization time-of-flight mass spectrometry (SELDI-TOF-MS) allowed the identification of 6 proteins able to distinguish primary from secondary cases with high sensitivity (115). Another proteomic study identified higher haptoglobin levels as a favorable serum biomarker for predicting long-term response to splenectomy in ITP, with a positive correlation with postoperative platelet count (110).

## Discussion and Future Perspectives

AIC secondary to CLL are a nice model of close intersection between cancer and autoimmunity. Both are the result of uncontrolled and dysregulated homeostatic mechanisms leading to aberrant proliferation and activity of specific cellular subsets with heterogeneous epiphenomena. Leukemic B-cells show impaired apoptosis, are unable to efficiently produce immunoglobulins, may function as antigen presenting cells, and release a variety of inflammatory cytokines leading to three main immune-related complications: infections, autoimmune diseases, and decreased immune-surveillance on secondary malignancies. These complications seem to correlate with advanced stage CLL and with poor prognostic markers. Moreover, CLL therapy may have an impact on their development.

**Figure 2:**
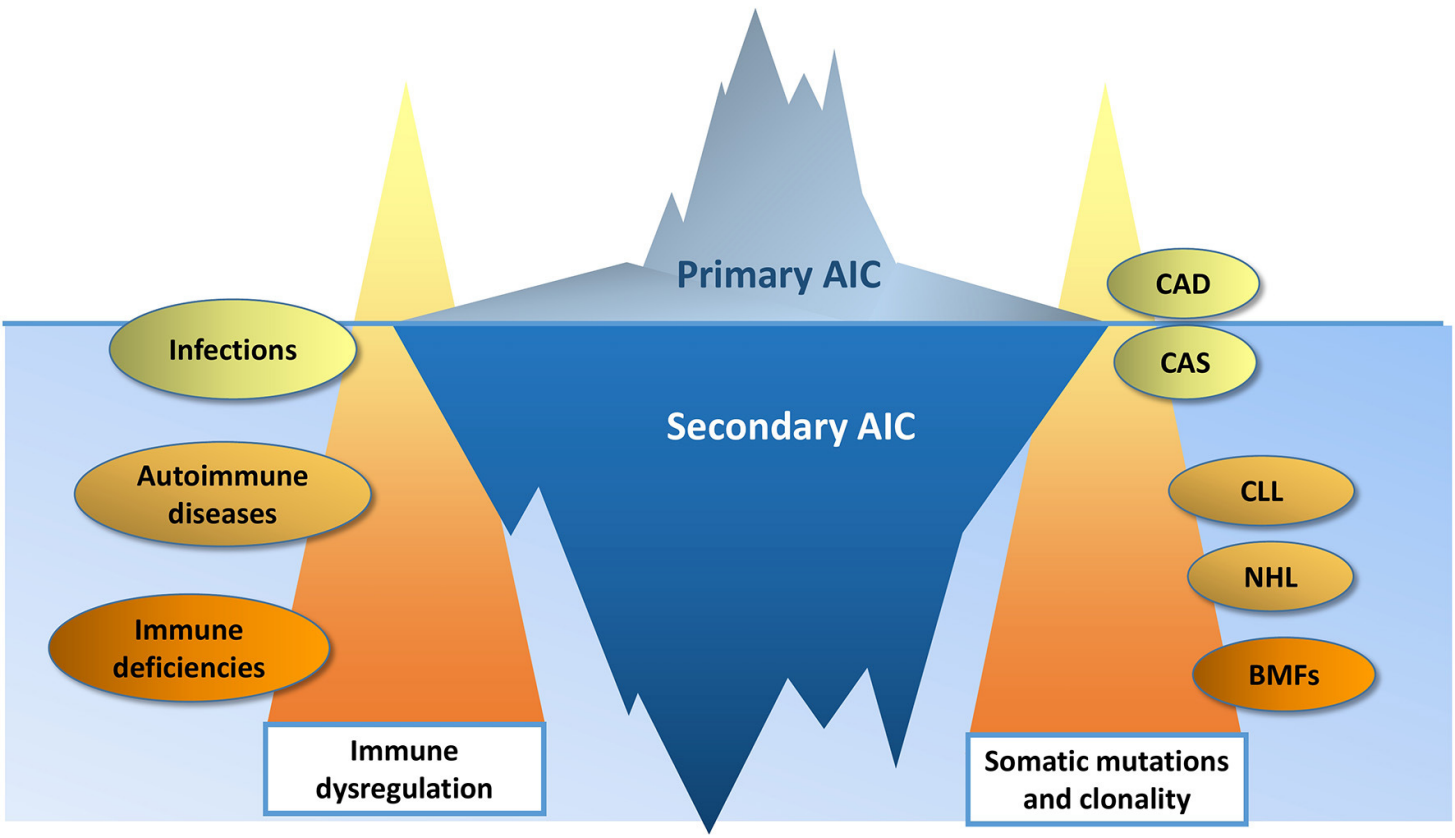
The changing border between primary and secondary autoimmune cytopenias (AIC). Immune dysregulation is more profound in AIC secondary to systemic autoimmune diseases and immune deficiencies, than in AIC secondary to infections. Likewise, a higher burden of somatic mutations is more typical of bone marrow failures (BMF) and lymphoproliferative disorders (chronic lymphocytic leukemia, CLL; non-Hodgkin lymphomas, NHL), than in cold agglutinin disease (CAD) and syndrome (CAS). The increasing availability of genomic testing will improve the diagnostic sensitivity, moving upward the border between primary and secondary AIC.

The genomic landscape of primary and secondary AIC is of particular interest, since the type and the depth of the immune response is likely under genetic control and it could be hypothesized that a predisposing genetic background correlates with a more profound immune dysregulation. Molecular studies performed so far, mainly focused on B-cell/autoantibodies characteristics and functioning, and on T cell aberrations: sterotyped B cells with specific IGHV and light chain configuration are involved in AIC development, clonal T cells, specifically CD8+ ones are present, and various cytokine genes polymorphisms may correlate with Treg/Th17 imbalance. Other experiences showed a dysregulation at the gene expression level as demonstrated by altered miRNA and lnRNA profiles in AIC cases compared to healthy subjects, but also in newly-diagnosed vs. chronic patients, and in the same patients in different tissues. Finally, proteomic studies reported differentially translated proteins in primary vs. secondary cases. In this regard, all the guidelines on AIC state that secondary causes should always be excluded. However, current workup relies mainly on laboratory, morphologic and imaging techniques that could be unable to disclose the presence of clonal disorders (Figure 2). In this context, the genetic/molecular characterization of AIC patients will probably increase our sensitivity in diagnosing secondary cases. This has been demonstrated in the recent paper on a pediatric Evans' population, where NGS/WES techniques revealed the presence of an underlying disease in 65% of cases, with important clinical/therapeutic implications. No data are available for adults, but for cAIHA, where a clonal lymphoid infiltrate is almost invariably present. This form is particularly difficult to distinguish from secondary cases. Berentsen and Colleagues proposed to differentiate cold agglutinin “disease” from “syndrome” basing on the absence or presence of a secondary cause. The demonstration that *MYD88* mutation is always absent and that *KMT2D* and *CARD11* ones are present in a proportion of cases, carry diagnostic, prognostic and therapeutic impact, further stressing the utility of molecular studies in AIC. Finally, there is growing evidence that AIC may evolve to overt clonal diseases of myeloid or lymphoid lineages and no predictors are available (138–141). This tempts to speculate about a model of “double clonality” unique for these forms, where either myeloid or lymphoid populations may undergo clonal expansion/selection. As a matter of fact, clonality and malignancy are distinct although overlapping concepts, and the evolution of a clonal disorder into an overt malignancy may require a long time, even longer than human lifespan. The immune system has a role in this process. However, it is not always clear whether it acts as an effector or spectator, and the exact molecular/genetic mechanisms and therapeutic implications have still to be disclosed.

## Author Contributions

BF and WB designed and wrote the review and participated to the final revision. All authors participated to the design of the review, literature revision, manuscript writing, and final revision for important intellectual content.

### Conflict of Interest

The authors declare that the research was conducted in the absence of any commercial or financial relationships that could be construed as a potential conflict of interest.
